# Immune mechanisms of protection against *Mycobacterium tuberculosis*‐centers

**DOI:** 10.3389/fimmu.2024.1429250

**Published:** 2024-10-08

**Authors:** Que Dang, Katrin Eichelberg, Nancy Vázquez-Maldonado, César Boggiano, Wolfgang W. Leitner, Lakshmi Ramachandra, Alison Deckhut-Augustine, Sarah W. Read

**Affiliations:** ^1^ Division of AIDS, National Institute of Allergy and Infectious Diseases, National Institutes of Health, Rockville, MD, United States; ^2^ Division of Microbiology and Infectious Diseases, National Institute of Allergy and Infectious Diseases, National Institutes of Health, Rockville, MD, United States; ^3^ Division of Allergy, Immunology, and Transplantation, National Institute of Allergy and Infectious Diseases, National Institutes of Health, Rockville, MD, United States

**Keywords:** *Mycobacterium tuberculosis*, TB vaccine, IMPAc-TB, TB immune responses, mechanisms of protection

Tuberculosis (TB), caused by *Mycobacterium tuberculosis* (*Mtb*), is one of the world’s leading infectious disease killers and is second only to COVID-19 deaths. TB has been found to be present in all countries and age groups. Although TB is a preventable and curable disease, an estimated 10.6 million people in 2022 developed TB worldwide. This included 5.8 million men, 3.5 million women, and 1.3 million children. Of these individuals, 1.3 million people died, including 167,000 people with HIV (1). Notably, TB is the leading cause of death among people living with HIV.

As one of the world’s largest funders of biomedical research on TB, the National Institute of Allergy and Infectious Diseases (NIAID) at the National Institutes of Health (NIH) has prioritized investing in TB to save lives. Specifically, NIAID supports and conducts basic, translational, and clinical research to better understand TB and expedite the development of innovative new tools and strategies to improve diagnosis, prevention, and treatment of TB.

In 2016, NIAID convened a workshop on “The Impact of *Mtb* Immune Evasion on Protective Immunity: Implications for TB Vaccine Design”. This workshop brought together global experts with the goal of defining immune mechanisms that could be targeted through novel research approaches and to inform vaccine design and immune therapeutic interventions for the prevention of TB. The meeting identified scientific gaps and influenced the initiative that led to the establishment of the Immune Mechanisms of Protection Against *Mycobacterium tuberculosis* Centers (IMPAc-TB) program (2). The overarching goal of the IMPAc-TB program is to understand the nature, location, and timing of immune responses required to prevent initial *Mtb* infection and to develop a comprehensive understanding of the immune responses required to prevent progression along the continuum of TB disease. Other goals of the IMPAc-TB program include understanding the effects of co-infections such as HIV on immune responses to *Mtb* infection or TB vaccination and improving the value of animal models in predicting TB vaccine efficacy in humans. These goals align with those of the NIAID Strategic Plan for Tuberculosis Research.

To accomplish these objectives, three contracts were awarded to multi-disciplinary research teams: HI-IMPAc-TB: Principal Investigators: Sarah Fortune, M.D. (Harvard); Henry Boom, M.D. (Case Western Reserve University); and JoAnne Flynn, Ph.D. (University of Pittsburgh); Phoenix: Principal investigator: Rhea Coler, Ph.D. (Seattle Children’s Research Institute); and Cascade: Principal investigator: Kevin Urdahl, M.D., Ph.D. (Seattle Children’s Research Institute) (3). The goals of the three centers can be viewed at IMPAc-TB.

**Figure 1:**
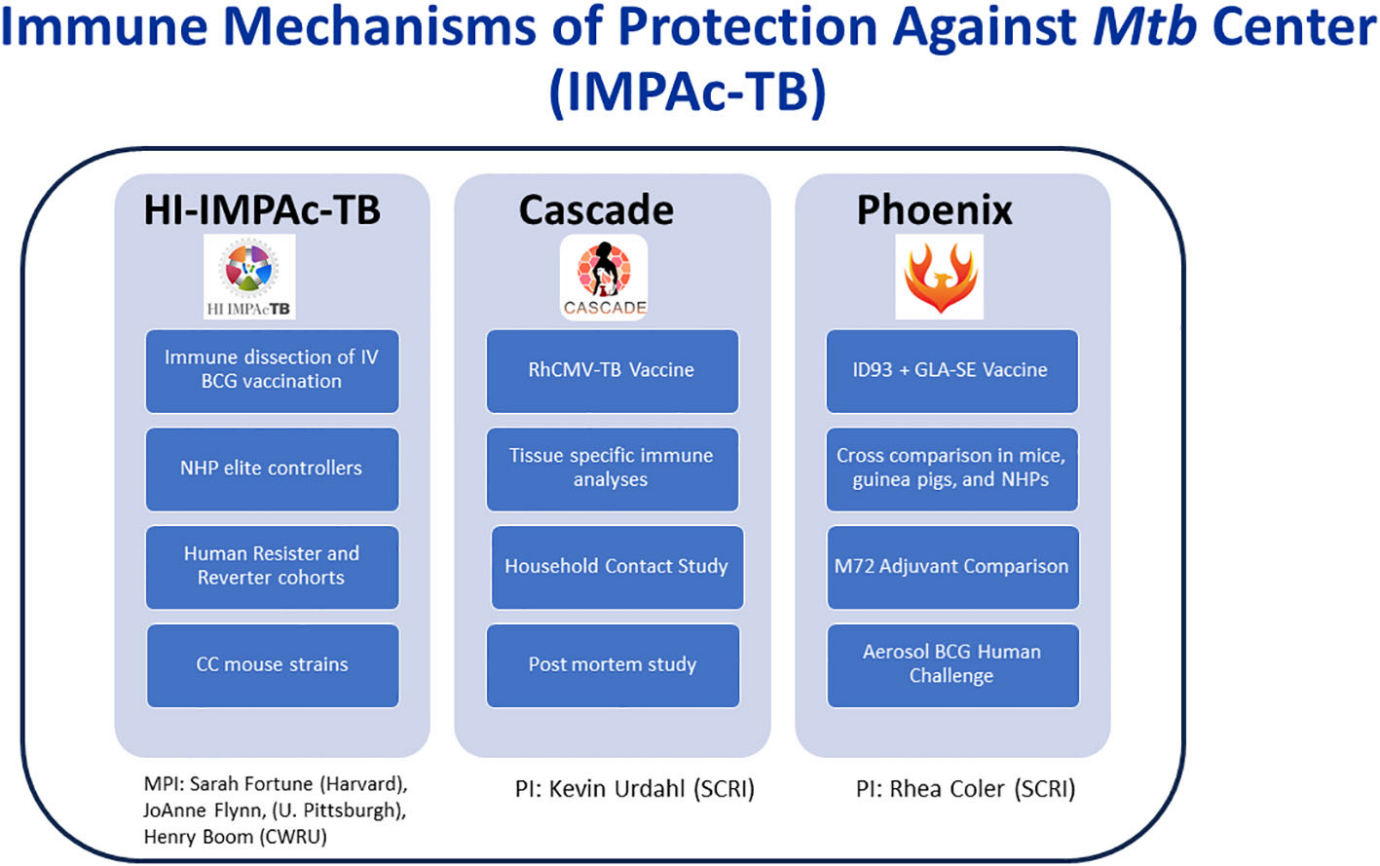
Highlighted research areas of the of the three awarded IMPAc-TB centers. MPI, multiple principal investigators; PI, principal investigator; CWRU, Case Western Reserve University; SCRI, Seattle Children’s Research Institute; HI-IMPAc-TB, Harmonized Investigation of the Immune Mechanisms of Protection Against TB.

**Figure 1** briefly illustrates the unique aspects of each IMPAc-TB center.

Although all three centers are pursuing distinct goals and addressing different research questions, collectively they conduct analyses on tissue-specific and systemic immune responses in small animals, nonhuman primates (NHPs), and humans to identify and to characterize the immunological space associated with protection and susceptibility to *Mtb*. Interestingly, the different approaches used by the centers lead to the identification of converging themes that may play a role in protection and/or susceptibility.

Other select highlights and contributions by the centers are as follows:

- Development of the first clinical trial employing a controlled human infection model to test the efficacy of a TB vaccine candidate. Participants will be challenged with aerosolized Bacille Calmette-Guerin (BCG). This clinical trial is expected to start at the end of 2024.
- Cross-species comparison to define immune mechanisms for the prevention of infection or disease using mice, including Collaborative Cross mice, guinea pigs, NHPs, and humans.
- Ultra-low dose challenge model in small animals and in NHPs.
- Back-translation, refinement, and validation of preclinical models with human data to improve their predictive value.
- Validation of newly developed guinea pig-specific immunological reagents to be offered to the field.
- Development of novel analytical frameworks for the integration of complex and multi-modal profiling data sets that will advance the identification of correlates of protection across species.

The subsequent articles in this issue of *Frontiers* exemplify additional work by the three centers.

The IMPAc-TB program is one of NIAID’s largest investments in TB vaccine research. Additionally, NIAID supports other TB vaccine-related programs such as Innovation for TB Vaccine Discovery (ITVD) and Advancing Vaccine Adjuvant Research for TB (AVAR-T). The 2024 NIAID Strategic Plan for Tuberculosis Research Update reaffirms the commitment of NIAID to accelerate basic and preclinical, translational, and clinical research and to expedite the development of innovative tools and new strategies to end the TB pandemic.

We hope you enjoy reading the articles in this special issue!
